# Influence of the Age of Free-Living Amoeba Cysts on Their Vertical Distribution in a Water Column

**DOI:** 10.3390/microorganisms12030474

**Published:** 2024-02-27

**Authors:** Zineb Fechtali-Moute, Sébastien Pomel

**Affiliations:** Université Paris-Saclay, CNRS BioCIS, 17 avenue des Sciences, 91400 Orsay, France

**Keywords:** free-living amoeba, cyst, age, water column, vertical distribution

## Abstract

Free-living amoebae (FLA) are widely distributed protozoa in both natural and artificial environments such as drinking water. In addition to the ability of all FLA to transport various pathogenic microorganisms, certain species, such as *Acanthamoeba* spp. or *Balamuthia mandrillaris*, have intrinsic pathogenic abilities and cause severe cerebral infections. Previous work has shown an enrichment of FLA cysts in biofilm developed in upper levels of Drinking Water Storage Towers (DWSTs), suggesting that differences in densities of FLA cysts may play a role in their unequal distribution in the water column. To evaluate this hypothesis, a model of a water column was created for this study and used to analyze the vertical distribution of cysts of the FLA *Acanthamoeba castellanii*, *Vermamoeba vermiformis*, and *Balamuthia mandrillaris* from 0 to 23 weeks. Interestingly, our data showed that the cysts of both *A. castellanii* and *V. vermiformis* were enriched in upper water levels during their aging. However, *B. mandrillaris* cysts were equally distributed in the water column during the entire study. These results show that, in addition to the role of water level variation in the DWST, some FLA cysts can become less dense during their aging, which contributes to their enrichment in upper water and therefore biofilm levels.

## 1. Introduction

Free-living amoebae (FLA) are unicellular eukaryotes that can be isolated from a wide range of habitats such as soil, rivers, and drinking water [1,2]. During their life cycle, FLA can alternate between two forms: a vegetative form, called a trophozoite, which produces pseudopods responsible for cellular movements and feeding, and a dormant and resistant form, called a cyst, which develops when environmental conditions are hostile for the amoeba [3,4].

Some of these FLA are qualified as amphizoic, implying that they are able to exist as free-living organisms in nature and can occasionally invade animal hosts and become pathogenic. These amphizoic FLA can cause serious ocular or cerebral pathologies. While ocular infections, called Amoebic Keratitis (AK), are caused by FLA from the genus *Acanthamoeba*, cerebral infections named Granulomatous Amoebic Encephalitis (GAE), *Balamuthia* Amoebic Encephalitis (BAE), and Primary Amoebic Meningoencephalitis (PAM) are caused by *Acanthamoeba* spp., *Balamuthia mandrillaris*, and *Naegleria fowleri*, respectively [4,5]. 

As several FLA have been described as resistant to disinfection methods used in drinking water, such as chlorine, ultraviolet irradiations, or ozonation [6,7,8], they can proliferate at different points in Drinking Water Networks (DWNs), such as distribution systems or storage towers. In particular, these protozoa can develop in the biofilms of DWN, where they will feed on bacteria and become a major source of amoeba-resisting microorganisms in drinking water [9], therefore posing a risk to human health. We have shown in a previous study conducted in three Drinking Water Storage Tanks (DWSTs) of a Drinking Water Network in the Parisian area (France), that FLA were enriched in the biofilms collected at the surface of the DWST, with an amoebal density (quantity of amoebae per mL) 10 times higher than at the bottom [2]. In this work, the amoeba *Acanthamoeba castellanii* was detected in each DWST, and FLA were isolated only in the cyst form. The unequal distribution of amoebae in the biofilm was hypothesized to be due to vertical variation of the water level in the DWST, called the tidal range, which would allow organic matter deposition and drying on the support at each immersion/emersion cycle and therefore promote cell adhesion and biofilm formation in the upper levels of the DWST. Apart from this hypothesis, the cell volumetric mass density of FLA cysts could be another factor, independent of biofilm formation, that contributes to their unequal distribution in the biofilm of the DWST. To further analyze this second hypothesis, a laboratory-scale water column mimicking a water reservoir was specifically developed in this study to analyze the vertical distribution of cysts of three different FLA species, i.e., *Acanthamoeba castellanii*, *Vermamoeba vermiformis*, and *Balamuthia mandrillaris*, as a function of their age. 

## 2. Materials and Methods

### 2.1. Chemicals

All chemicals were supplied by Merck Laboratories (St-Quentin-Fallavier, France), except for Fetal Bovine Serum (FBS; Thermo-Fisher, Villebon-sur-Yvette, France).

### 2.2. FLA Culture

*Acanthamoeba castellanii* (ATCC 30010 strain) and *Vermamoeba vermiformis* (ATCC 50237 strain) were grown axenically in PYG medium (ATCC medium 712) and PYNFH medium (ATCC 1034), respectively, following the procedure described in [10]. The PYG is composed of 2% (*w*/*v*) peptone, 0.1% (*w*/*v*) yeast extract, 100 mM glucose, 4 mM MgSO_4_, 400 µM CaCl_2_, 3.4 mM sodium citrate, 2.5 mM Na_2_HPO_4_, 2.5 mM KH_2_PO_4_, and 50 µM Fe (NH_4_)_2_(SO_4_)_2_, with a pH of 6.5, and the PYNFH medium is constituted of 1% (*w*/*v*) bacto-peptone, 1% (*w*/*v*) yeast extract, 0.1% (*w*/*v*) RNA type VI from torula yeast, 34 µM folic acid, 1.5 µM hemin, 3.5 mM Na_2_HPO_4_, 2.7 mM KH_2_PO_4_, and 10% (*v*/*v*) FBS, with a pH of 6.5. 

*Balamuthia mandrillaris* (ATCC 50209 strain) was cultured in RPMI-1640 medium supplemented with 10% FBS (complete RPMI-1640 medium), penicillin (100 U/mL), and streptomycin (100 µg/mL; Fisher Scientific, Illkirch, France) on a monolayer of Vero cells (ATCC; Manassas, VA, USA), as previously reported [11]. Briefly, culturing required two steps: the first step consisted of subculturing the Vero cells at 1 × 10^5^ cells/mL in complete RPMI-1640 medium for three days, and in the second step, the culture medium was changed gently to avoid detachment of the Vero adherent cells, and it was replaced by RPMI-1640 complete medium containing *B. mandrillaris*.

FLA *A. castellanii* and *V. vermiformis* were grown at 27 °C, while Vero cells and *B. mandrillaris* were cultured at 37 °C in the presence of 5% CO_2_. All FLA were cultured in the dark without shaking, with an initial cell density of 5 × 10^4^ amoebae/mL, and were passed twice a week under these conditions. 

### 2.3. Encystment

As previously described [12], 15 mL of trophozoites at 1 × 10^6^ amoebae/mL were centrifuged at room temperature at 3000× *g* for 10 min, further resuspended, and washed three times in Neff buffer containing 0.1 M KCl, 8 mM MgSO_4_, 0.4 mM CaCl_2_, 1 mM NaHCO_3_, 20 mM Tris-HCl, pH 8.8 [13]. Cysts were generated after incubation of amoebae in 30 mL of Neff buffer at 27 °C for five days. Mature cysts were further selected after treatment at room temperature for 10 min with 0.5 % (*w*/*v*) SDS (Sodium Dodecyl Sulfate), eliminating trophozoites and pseudocysts, which are sensitive to SDS. By comparing the number of FLA before (total FLA) and after (mature cysts) SDS treatment, we observed that ~95-100% of *A. castellanii* and *V. vermiformis* were encysted, while only ~70% of *B. mandrillaris* were transformed into cysts. Mature cysts were further washed three times in PAS buffer (ATCC medium 1323) containing 1 mM Na_2_HPO_4_, 1 mM KH_2_PO_4_, 16 µM MgSO_4_, 27 µM CaCl_2_, 2 mM NaCl, with centrifugation at 3000× *g* for 15 min between each washing step. At this step, the cyst age was defined as 0 weeks, corresponding to the starting point of the study. The cysts were then stored at 4 °C in PAS buffer for 23 weeks, corresponding to the whole duration of the study, and used for further experiments at each specified time point. The PAS buffer was changed every four weeks by centrifuging the cyst suspensions at 3000× *g* for 15 min at 4 °C. Before adding the cysts to the laboratory-scale model of the water column, the cysts were centrifuged at 3000× *g* for 15 min, resuspended in distilled water pre-filtered through a 0.22 µm porosity filter (Sartorius Stedim Biotech, Aubagne, France), and passed through a 25G needle (Terumo, Rueil-Malmaison, France) to avoid any clustering.

### 2.4. Model of Water Column

**Figure 1:**
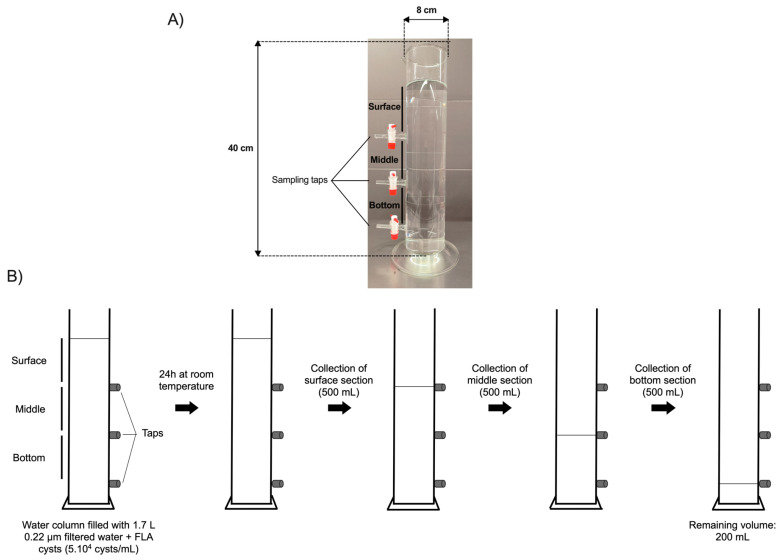
Laboratory scale model of a water column and schematic representation of the water sampling method. (**A**) Photography of the laboratory-scale model of the water column equipped with three taps, allowing water sampling in distinct sections: surface, middle, and bottom. (**B**) Schematic representation of the water sampling method in the surface, middle, and bottom sections.

A laboratory-scale model of a water reservoir was specifically designed for this study by the VERRE SCIEN-TECH facility of the Faculty of Pharmacy—Paris Saclay University (Figure 1A). The model of the water column is a glass cylinder of 40 cm height and 8 cm diameter, thus containing a volume of up to 2 L. The model was equipped with three taps installed at equal distances from each other, thus defining three sections: surface, middle, and bottom, allowing the collection of an equal volume of 500 mL in each section. The three taps were installed at the bottom of each section. 

### 2.5. Sampling

The cysts of *A. castellanii*, *V. vermiformis*, or *B. mandrillaris*, aged 0 to 23 weeks, were added to the water column containing 1.7 L of distilled water pre-filtered through a 0.22 µm porosity filter (Sartorius Stedim Biotech, Aubagne, France), in order to mimic, in a laboratory-scale model, the conditions of water storage in a DWST, with a maximal volume of 3 mL to reach a final cellular density of 5 × 10^4^ cysts/mL. The water column was further covered with parafilm and a cap of aluminum foil (Figure 1B).

After 24 h of incubation at room temperature without agitation, 500 mL of water samples were collected from each section in the following order to minimize water mixing between sections as much as possible: surface first, then middle, then bottom. As the tap in the bottom section could not be installed at the lowest level of the column for technical reasons, it was placed 4 cm above the base of the water column, leaving a volume of 200 mL below the lowest tap (remaining volume; Figure 1B). Following water sampling in each section, samples were centrifuged at 3000× *g* for 15 min at room temperature. The pellets were resuspended in 10 mL of pre-filtered distilled water, and the cysts were counted in triplicates using a Malassez counting chamber (Fisher Scientific, Illkirch, France). From the cyst quantities initially added to the water column and further counted in each section, the proportion of cysts that were not taken into account in the study, either left in the remaining volume below the lowest tap or adhered to the vessel, was estimated to be 11% to 16% of the total cyst population. Therefore, the proportion of FLA cysts in suspension between the surface and bottom sections that was analyzed in the current work represents a vast majority of the cyst populations initially added to the water column, i.e., 84% to 89% of the cysts.

### 2.6. Statistical Analyses

The statistical analyses of the results were carried out using the Kruskal–Wallis non-parametric test (*p* < 0.0001), followed by the uncorrected Dunn’s multiple comparison test (*p* < 0.05), using GraphPad Prism software (version 9.3.1; San Diego, CA, USA).

## 3. Results

The aim of this work was to compare the vertical distribution of cysts from three different FLA species, namely *Acanthamoeba castellanii*, *Vermamoeba vermiformis*, and *Balamuthia mandrillaris*, in a water column as a function of their age, from 0 to 23 weeks. 

**Figure 2:**
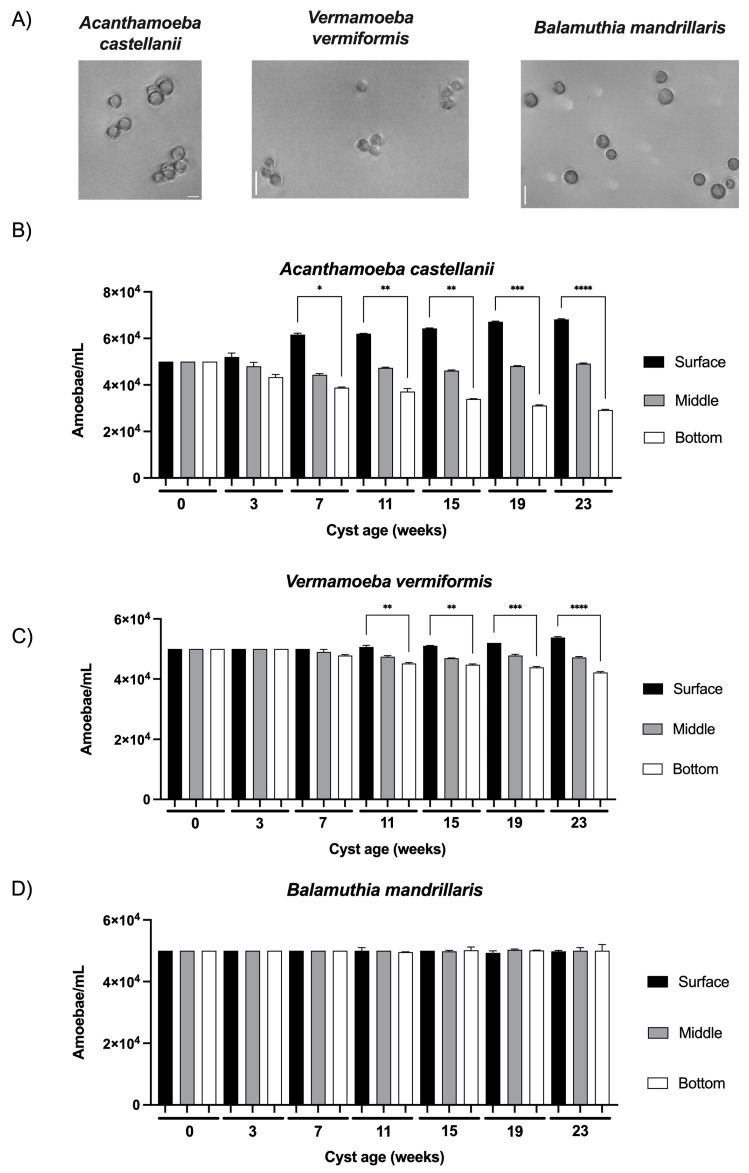
Observation and vertical distribution of FLA cysts in a water column model as a function of their age. (**A**) Images of cysts of *Acanthamoeba castellanii* (**left**), *Vermamoeba vermiformis* (**middle**), and *Balamuthia mandrillaris* (**right**) obtained at the age of 0 weeks in phase contrast using an inverted microscope Leica DMi8. Scale bar: 20 µm. Vertical distribution of (**B**) *Acanthamoeba castellanii*, (**C**) *Vermamoeba vermiformis*, (**D**) *Balamuthia mandrillaris* in the laboratory-scale model of the water column. The results correspond to the means of three independent countings ± SD. * 0.05 ≤ *p* < 0.01, ** 0.01 ≤ *p* < 0.001, *** 0.001 ≤ *p* ≤ 0.0001, **** *p* < 0.0001.

Just after their formation at the age of 0 weeks, the cysts, which were selected based on their resistance to SDS treatment [12], developed a cell wall with characteristic layers typical of mature cysts of the three FLA species studied in the current work (Figure 2A). At this initial time point, the cysts of *A. castellanii*, *V. vermiformis*, and *B. mandrillaris* were equally distributed in the three sections, namely surface, middle, and bottom, with an amoebal density of 5 × 10^4^ amoeba/mL after a 24 h incubation in the water column (Figure 2B–D). Although no significant difference was observed in cyst morphology or volume within each species of the three FLA studied for the whole duration of the study, cyst size varied substantially between FLA species: *A. castellanii* from 14 to 23 µm, *V. vermiformis* from 9 to 13 µm and *B. mandrillaris* from 11 to 17 µm (Figure 2A). Additionally, several variations in vertical distribution were noticed according to cyst age: at the age of three weeks, the cysts of *A. castellanii* showed a tendency to be more abundant at the surface compared to the bottom (Figure 2B). This tendency was confirmed when older cysts showed significant differences in amoebal density between the surface and the bottom section at seven weeks and then increased with time: *A. castellanii* cysts were 1.6 to 2.3 times more abundant in the surface section compared to the bottom from the age of 7 weeks to 23 weeks, respectively (Figure 2B). Concerning the cysts of *Vermamoeba vermiformis*, the same tendency of unequal distribution between surface and bottom sections as a function of cyst age was observed, but with a lower intensity. A significant increase of 12% of *V. vermiformis* cysts between surface and bottom sections started at 11 weeks, and this difference gradually increased to 27% until 23 weeks (Figure 2C). From 0 to 23 weeks, the significant increases in cyst abundance at the surface section were also accompanied by a significant decrease of 41% and 16% for *A. castellanii* and *V. vermiformis*, respectively, at the bottom section (Figure 2B,C). Conversely, no significant differences were observed in the middle section, either for *A. castellanii* or *V. vermiformis*, as a function of cyst age during the 23 weeks of the study (Figure 2B,C). Furthermore, no variation in *B. mandrillaris* cyst vertical distribution was observed as a function of their age (Figure 2D): similar cyst concentrations were determined at the three levels of the water column during the whole duration of the study. 

## 4. Discussion

In the current work, we observed an enrichment of *A. castellanii* and *V. vermiformis* cysts, but not *B. mandrillaris* cysts, in the surface section of the water column during a period of 23 weeks of cyst aging, suggesting a variation in the volumetric mass density of the cysts of these FLA species as a function of their age. To our knowledge, three main hypotheses may explain the differences in FLA cyst distribution observed in the present work. 

Firstly, *Acanthamoeba castellanii* has been described to gradually lose water during encystment [14]. If this process persists during cyst aging after its formation, it would have induced an increase in its volumetric mass density, as more material would be accumulated in less space and thus lead to cyst shrinkage, in direct opposition to the results observed in our current work. Our results indicate that water loss would not be the factor inducing an enrichment of *A. castellanii* and *V. vermiformis* cysts in upper water levels as a function of their age.

Secondly, one could hypothesize that these differences could be attributed to variations in cyst wall composition between FLA species. Indeed, the cyst wall of the three FLA studied in the present work are different in terms of structure and biochemical composition. At the ultrastructural level, *Acanthamoeba* spp. and *Vermamoeba* spp. cyst walls are composed of two layers [14,15], while *Balamuthia mandrillaris* contains a supplementary coat [16]. The cyst wall of *B. mandrillaris* is presumably composed of proteins and cellulose, with proportions that remain to be determined [17,18]. Additionally, the cyst wall of *A. castellanii* is mainly composed of proteins and carbohydrates, especially cellulose [19,20,21], while the cysts of *V. vermiformis* are composed of more than 64 % proteins and only 4.2% cellulose [22]. However, as the volumetric mass density of cellulose-containing molecules increases with cellulose content [23], an increase in cellulose content in FLA cyst walls during their aging would not lead to a displacement to upper water levels. Moreover, as cellulose is the main cyst wall component in *A. castellanii* but not in *V. vermiformis*, where an enrichment in surface water levels was also observed for older cysts, the presence of this hydrophilic polymer in cyst walls does not seem to be the factor influencing FLA cyst volumetric mass density during their aging. While the hydrophobicity of proteins composing FLA cyst walls could also be considered, it is improbable that *A. castellanii* or *V. vermiformis* would synthesize more proteins, specifically hydrophobic proteins, on their surface during cyst aging, as FLA cysts have low metabolism [24,25]. Moreover, if the SDS treatment used in our method to select mature cysts, as previously described in [12], would have introduced artifacts in cyst wall composition, these changes would probably not have evolved further as a function of FLA age. Therefore, this treatment is presumably not a main factor influencing the floatability of cysts as a function of their age. 

During encystment, *A. castellanii* degrades some organelles, including digestive vacuoles or mitochondria, as well as several macromolecules such as proteins, phospholipids, or glycogen [14]. These degradation products could then be used as a source of energy in the FLA cyst, which is metabolically dormant, leading to a decrease in dry weight during cyst formation. Accordingly, in mammalian cells, different volumetric mass densities have been measured during the cell cycle of a cell line, as well as between cell lines, probably because of different quantities of biochemical components such as proteins or nucleotides [26,27,28]. Therefore, a third hypothesis, presumably the most plausible, could be emitted in the current study, assuming that macromolecule degradation would continue after cyst formation, during their aging, leading to a decrease in their dry weight as well as their volumetric mass density, and therefore to their redistribution to upper water levels. In addition to this possibility, within the cyst wall of *A. castellanii*, as well as *V. vermiformis*, the endocyst is separated from the ectocyst by a space that could contribute to cyst floating in upper levels of the water column, in opposition to *B. mandrillaris*, where the cyst layers are more compact [12,17]. According to our data, this process of cyst maturation after encystment would occur in *A. castellanii* and, to a lesser extent, in *V. vermiformis*, but not in *B. mandrillaris*, where no variation of FLA cyst distribution was observed as a function of their age. Therefore, in addition to the role of the tidal range in the enrichment of FLA in the biofilms collected on the surface of DWST [2], this phenomenon of volumetric mass density decrease as a function of cyst age would also contribute, independently of the biofilm formation, to the increase in some FLA species at the surface water level and thereafter in the biofilm. In particular, as *A. castellanii* and *V. vermiformis*, are accumulated at surface water levels as a function of their age, these FLA species should be particularly further monitored on the surface of DWSTs, as they could constitute a reservoir of pathogenic microorganisms and therefore pose a potential risk to human health.

In future works, as the FLA cyst age appears to influence cyst distribution in a water column, the analysis of biochemical compositions of young and old cysts of different FLA species would allow us to examine the process of FLA cyst maturation after encystment in more detail and determine its role in cyst distribution in a water column. Furthermore, the comparison of the distribution of young cysts of *A. castellanii* in a model of water reservoir presenting a tidal range with the dispersion of old cysts of the same FLA species in a water column without a tidal range would allow us to determine the relative importance of the tidal range and FLA cyst age in the distribution of cysts in a DWST.

## Data Availability

Data are contained within the article.
